# The Role of Perceived Benefits in Buffering Gastrointestinal-Symptom Burden Among Post-Operative Colorectal Cancer Patients: A Six-Month Longitudinal Study

**DOI:** 10.3390/cancers17172934

**Published:** 2025-09-08

**Authors:** Ming-Wei Chang, Ashley Wei-Ting Wang, Cheng-Shyong Chang

**Affiliations:** 1 Department of Psychiatry, Far Eastern Memorial Hospital, New Taipei City 220216, Taiwan; 2 Department of Psychology, Soochow University, Taipei 111002, Taiwan; 3 Division of Hematology-Oncology, Chang Bing Show Chwan Memorial Hospital, Changhua 505029, Taiwan

**Keywords:** gastrointestinal symptoms, benefit finding, buffer, health-related quality of life, colorectal cancer

## Abstract

**Objectives:** The goal of this longitudinal study was to explore how gastrointestinal-symptom distress and benefit finding together influence health-related quality of life (HRQOL) over time in colorectal cancer (CRC) survivors. Colorectal cancer (CRC) survivorship may differ from other cancer types due to the persistent burden of gastrointestinal symptoms. The highly visible and socially sensitive nature of these sequelae may influence the trajectory of benefit finding compared with other cancers. Specifically, we explored whether increases in symptom distress predicted declines in physical and mental HRQOL whether benefit finding was directly related to or buffered the negative impact of symptom distress on HRQOL. **Methods:** Participants were 73 Taiwanese women and men who underwent surgery for CRC. Using a three-time-point, multilevel framework, participants were assessed at 1, 3, and 6 months after surgery. Hierarchical linear modeling was used to investigate whether gastrointestinal-symptom distress and benefit finding covary over time with HRQOL. **Results:** The results indicated that increases in gastrointestinal-symptom distress were linked to declines in both physical and mental HRQOL. Benefit finding had no direct association with HRQOL but significantly moderated the symptom-HRQOL relationship, weakening the negative impact of symptom distress among those with higher benefit finding. **Conclusions:** Rises in a CRC survivor’s gastrointestinal-symptom distress went hand-in-hand with drops in both physical and mental HRQOL. This study adds to the literature in that benefit finding has a favorable effect on cancer adjustment when patients face higher symptom distress after the surgery and treatment. Further implications on possible mechanisms were discussed.

## Simple Summary

This study followed 73 Taiwanese colorectal cancer (CRC) patients for six months after surgery to understand how ongoing gastrointestinal symptoms and positive psychological changes (known as *benefit finding*) affect their quality of life. The findings showed that when patients experienced more distress from gastrointestinal symptoms, their physical and mental quality of life declined. However, those who were better able to find positive meaning in their cancer experience were less affected by these symptoms. While benefit finding did not directly improve quality of life, it helped protect patients from the negative impact of symptom distress. The study highlights the potential role of benefit finding in helping CRC survivors adjust after surgery.

## 1. Introduction

Colorectal cancer (CRC) is a major public health concern in Taiwan and globally. In Taiwan, colorectal cancer (CRC) has consistently been the most commonly diagnosed cancer in both men and women, with 17,643 new cases reported in 2022. It also ranks among the top three causes of cancer-related deaths [1]. Advances in early detection and treatment have significantly improved survival, with a current 5-year survival rate of approximately 65% [1], rising to 90% if diagnosed early [2]. As the CRC survivor population grows, so do healthcare costs, underscoring the need to address quality-of-life issues in the post-treatment phase.

Treatment- and disease-induced physical symptom distress is highly prevalent among individuals with CRC and remains a major contributor to reduced quality of life [3,4,5]. Notably, many survivors continue to experience both acute (≤3 months) and chronic (≥6 months) symptoms even after successful tumor eradication [6,7,8]. Among these, *gastrointestinal symptoms* are especially burdensome, often disrupting daily functioning and significantly impairing overall HRQOL [9]. Frequently reported gastrointestinal symptoms include increased bowel frequency, persistent bowel movements, perianal skin ulceration, abdominal distension, abdominal pain, vomiting, and a sensation of incomplete evacuation [7,8,9,10]. These symptoms not only compromise physical well-being but also impose considerable emotional and social burdens, often resulting in diminished HRQOL [3,4,5,9]. HRQOL is a multidimensional construct encompassing both physical and mental health, typically assessed using the physical component summary (PCS) and mental component summary (MCS) scores [11]. Given the profound impact of gastrointestinal symptoms on both domains of HRQOL, identifying their predictive value for PCS and MCS outcomes is essential for guiding supportive care and survivorship interventions in the CRC population.

Despite the negative sequelae, survivors also often express positive changes as a result of the cancer experience [12]. *Benefit finding* refers to positive changes in perceptions, thoughts, and behaviors that individuals recognize following stressful experiences, including cancer diagnosis and treatment [13]. Cancer survivors often report a shift in priorities, with more focus on relationships, personal growth, and life appreciation, and less on minor concerns [14]. Although research on benefit finding has largely focused on breast cancer, CRC populations also commonly report positive psychological changes shortly after diagnosis [15,16,17]. Literature highlights benefit finding as a potential psychological resource that can mitigate the adverse impact of cancer-specific stressors on quality of life by fostering adaptive coping mechanisms, enhancing resilience, and providing emotional relief [18,19]. Studies suggest that individuals who report higher levels of benefit finding experience less psychological distress and better emotional adjustment, even in the presence of ongoing physical symptoms [20,21].

CRC survivorship is less frequently examined than survivorship in other types of cancer, such as breast or prostate cancer [22,23]. CRC survivors often contend with gastrointestinal and stoma-related symptoms—such as bowel dysfunction, stoma care needs, and fatigue—that can disrupt daily routines, diet, and social participation [24]. The visibility and social sensitivity of these symptoms may influence the trajectory of benefit finding differently from the body-image concerns more typical of breast cancer and the sexual concerns emphasized in prostate cancer [17], supporting the need for CRC-specific survivorship research.

While benefit finding moderates psychological distress, its buffering effect on the relationship between cancer-specific physical symptoms and HRQOL remains under-investigated [12,18,20,25,26,27]. Given the unique symptomatology and survivorship experiences associated with CRC, it is crucial to examine whether benefit finding serves as a significant protective factor, buffering survivors against the adverse consequences of persistent gastrointestinal symptoms. Benefit finding buffers the impact of symptom burden on quality of life through mechanisms including improved reappraisal of bodily sensations, greater self-efficacy in symptom management, and enhanced resilience that disrupts the symptom–distress–QoL pathway [28,29]. Clarifying this moderating role could inform targeted psychosocial interventions designed to enhance survivors’ HRQOL by promoting adaptive psychological responses to ongoing symptom distress.

Therefore, this study aimed to (1) investigate how cancer-specific symptoms predict general HRQOL, encompassing both physical (PCS) and mental (MCS) components, among CRC survivors, and (2) evaluate whether benefit finding moderates these predictive relationships. Further, it has been suggested that perceiving meaning in cancer is an ongoing process [30,31,32], and so are physical symptoms. We extend the literature by examining how within-person fluctuations in symptoms and benefit finding predict HRQOL over time. This longitudinal, repeated-measures study examines how within-person changes in cancer-related gastrointestinal symptoms and benefit finding jointly predict HRQOL over the six months following CRC surgery, thereby addressing temporal dynamics that previous between-person research could not capture.

## 2. Methods

### 2.1. Participants and Procedures

Patients who underwent CRC surgery at a medical center in Taiwan were invited to participate during hospitalization, within one month post-surgery. Participants were eligible if they (1) were aware of the diagnosis of CRC, (2) did not have a history of other cancer, and (3) had no psychiatric history. Written informed consent was obtained from all the participants before the beginning of the study. The first assessment (T1) was conducted as soon as feasible after surgery (within 30 days), the second (T2) approximately three months later, and the third (T3) about six months after T1. T1 was conducted during hospitalization, while T2 and T3 were completed either during follow-up clinic visits or by mail. The study was conducted in accordance with ethical guidelines, and all procedures were approved by the Institutional Review Board of the Hospital.

### 2.2. Measures

All the other instruments were administered at all time points.

For cancer-specific physical symptoms, we focused on *gastrointestinal symptoms* among treatment- and disease-related physical symptoms because they are among the most prevalent and burdensome complaints in individuals treated for CRC [33]. Surgical resection, often involving partial removal of the colon or rectum, along with adjuvant therapies such as chemotherapy or radiotherapy, frequently disrupts normal gastrointestinal function. We developed seven items that capture the most frequently reported gastrointestinal symptoms experienced by CRC survivors, including increased frequency of bowel movements, consecutive bowel movements, perianal skin ulceration, abdominal bloating, abdominal pain, vomiting, and the sensation of incomplete bowel evacuation. The gastrointestinal symptom distress scale was newly developed for this study, drawing on clinical observations and reports of the most frequently experienced gastrointestinal complaints among CRC survivors, with reference to established measures including the EORTC QLQ-C30 [34], QLQ-CR29 [35], MSKCC Bowel Function Instrument [36], FACT-C [37], and LARS [38].

Participants were asked to rate the level of distress caused by each symptom. The five response options ranged from 0 (*not at all*) to 4 (*extremely distressing*). We conducted a principal-components analysis with Promax rotation at all time points. A single factor emerged (eigenvalue > 1), accounting for 61.9–67.2% of the variance across T1–T3, and the scree plot confirmed a one-factor solution. All items loaded ≥0.40 except item 6 (“vomiting”), which loaded <0.30 and reduced internal consistency (Cronbach’s α); therefore, it was removed. Cronbach’s α for the final 6-item gastrointestinal symptom distress scale were 0.79, 0.88, and 0.74 at T1, T2, and T3, respectively. We did not include other common symptoms of cancer survivorship, such as fatigue or sleep disturbance, because these are nonspecific sequelae shared across cancer types and may reflect broader psychosocial or systemic factors. By focusing on gastrointestinal symptoms, the scale captures the distinctive functional disruptions most directly tied to surgical and adjuvant therapies for CRC, thereby enhancing its specificity and clinical relevance for this population.

Benefit finding was measured by the positive meaning subscale of the Positive Meaning and Vulnerability Scale [14]. The scale is specifically designed for assessing perceived meaning (i.e., benefit finding) and vulnerability in response to cancer. The positive meaning subscale was chosen because of its specificity to cancer and its brevity for clinical populations. It was developed using mixed methods that comprised the literature review, focus groups with cancer survivors, and the clinical experience of the authors to assess key domains of change in outlook following cancer. The Perceived Meaning Scale consists of 6 items (e.g., “Surviving breast cancer has changed my outlook on life”) which assess changes in priorities, daily activities, relationships, and perspectives. The instruction was, “Indicate for each of the statements below the degree to which you believed your outlook had changed in each way as a result of breast cancer.” Responses were scored on a five-point Likert scale (1 = not at all, 5 = very much). In this study, the internal consistencies (α) of the meaning scores were 0.77, 0.81, and 0.86 for T1, T2, and T3, respectively.

HRQOL was measured by the SF-36 Health Survey (SF-36) [11]. SF-36 measures physical and psychosocial aspects of HRQOL on eight multi-item dimensions, covering physical functioning, role-physical, bodily pain, general health, vitality, social functioning, role-emotional, and mental health. The first four dimensions cover physical health (PCS), and the other four are concerned with mental health (MCS). The norm-based scoring for SF-36 is used; total PCS and MCS scores range from 0 to 100, with higher scores indicating better HRQoL. The Cronbach’s α coefficients in the present sample for the PCS and MCS were 0.89, 0.87, 0.91, and 0.88, 0.89, 0.90 for T1-3, respectively.

### 2.3. Statistical Analysis

Given that the central focus of our analyses is the extent to which within-person changes in gastrointestinal symptoms and benefit finding predict changes in HRQOL, we employed multilevel modeling using Hierarchical Linear Modeling (HLM) [39]. The central focus of our analyses concerns the extent to which within-person change in gastrointestinal symptoms and benefit finding predicts change in HRQOL. This approach captures individual change over time, enabling the detection of complex change trajectories [40]. The HLM equations are estimated using restricted maximum likelihood estimation procedures [40,41].

The study was a two-level design, in which three waves of assessments were nested within each participant. The outcome measure, HRQOL (i.e., PCS and MCS), and the predictor measures, gastrointestinal symptoms and benefit finding (both grand mean centered), and the interaction, were within-person variables. Because time since surgery may have an effect on HRQOL, our within-person level model takes into account the main effects of time since surgery. Based on prior research, age, gender, economic status (monthly family income), and cancer stage were included as between-person covariates [33], with all continuous variables (except gender) grand-mean centered.

Before conducting the HLM analyses, we used the intraclass correlation (ICC) [40] to assess the extent to which total variance in the outcome variables and predictors is due to fluctuations over time. The ICC reflects the proportion of total variance in an outcome that is attributable to differences between individuals, relative to fluctuations within individuals over time. ICCs close to 0 indicate substantial within-person variability, suggesting that scores fluctuate over time rather than remain stable. This highlights the importance of accounting for change over time, not just between-person differences, in longitudinal research.

If any interaction term was significant, the interaction was plotted following the procedure illustrated by Preacher, Curran [42], and conditional values of moderators were set at 1 *SD* above and below the mean.

Restricted maximum likelihood accommodates unbalanced longitudinal designs (i.e., unequal numbers), thereby using all available observations without ad hoc imputation [41]; thus, participants who discontinued before the last wave (73 enrolled; 56 at final assessment) still contributed their observed data to the fixed and random effects. In HLM, missing values are permitted for Level-1 outcomes and predictors. Our data contain no missing values on level-2 variables.

## 3. Results

### 3.1. Sample Demographics

Patients’ ages ranged from 28 to 72 years, with a mean of 50.2 years (*SD* = 10.76). Participants were diagnosed with stage 1 (*n* = 42), stage 2 (*n* = 11), stage 3 (*n* = 15), and stage 4 (*n* = 5) CRC. Participants included 26 males and 47 females; 58.6% held a university degree or higher; 77.2% were married; and 52.1% reported a household income below $2000/month. Among the participants, 64.4% had colon cancer and 35.6% had rectal cancer, while 54.3% received chemotherapy.

### 3.2. Descriptive, ICC, and Correlation Analyses

Average scores across time on all variables are reported in Table 1. The intraclass correlations (ICC) for PCS and MCS were 0.24 and 0.48, respectively, indicating that less than half of the variance was due to differences between individuals, while the majority (76% and 52%) reflected within-person fluctuations over time. The substantial within-person variability supports the rationale for examining changes over time in our analyses. The ICCs for gastrointestinal symptoms and benefit finding were 0.38 and 0.37, respectively, indicating that most of the variability (62% and 63%) was due to within-person fluctuations. This further supports the rationale for examining changes over time in our analyses.

At T1, T2, and T3, gastrointestinal symptoms and benefit finding were not significantly correlated, *r* = 0.20, 0.06, and 0.11 (*ps* = 0.10, 0.60, and 0.34), respectively. PCS and MCS were not correlated (*r* = −0.17 and 0.05, *ps* = 0.15 and 0.71), except for T3 (*r* = 0.43, *ps* < 0.001). Gastrointestinal symptoms were unrelated to PCS (*r* = −0.17 and −0.18, *ps* = 0.15 and 0.14), except for T3 (*r* = −0.46, *p* < 0.001). On the contrary, gastrointestinal symptoms were unrelated to MCS (*r* = −0.17 and −0.18, *ps* = 0.14 and 0.13), except for T1 (*r* = −0.38, *p* = 0.001). Benefit finding was unrelated to PCS and MCS at all interviews (*r*s = −0.17 to 0.004), except for marginal correlations with MCS at T1 and T3 (*r*s = −0.21 and −0.21, *ps* = 0.071 and 0.078).

Age was positively correlated with MCS (*r* = 0.32 to 0.37, *p* = 0.001 to 0.007), but negatively correlated with gastrointestinal symptoms (*r* = −0.23 to −0.43, *p =* 0.05 to *p* < 0.00). Gender, marriage status, and job status were unrelated to any study variables. None of the medical variables (disease stage, type of surgery, receipt of adjuvant therapy) was associated with the study variables.

### 3.3. HLM Model for PCS

Table 2 (first column) presents a summary of the results of the analysis predicting PCS scores, controlling for age, gender, income, and cancer stage. Income was positively associated with PCS, such that higher income corresponded to higher PCS.

**Figure 1:**
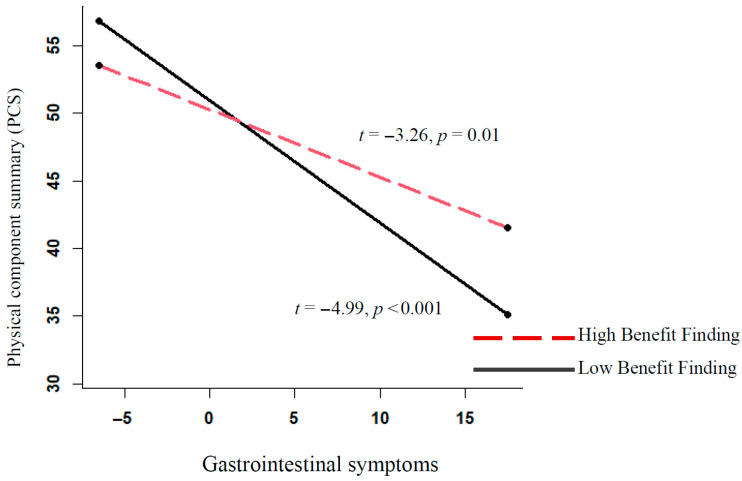
Moderation effect of benefit finding on gastrointestinal symptoms and PCS score. Depicted are slopes for 1 standard deviation above and 1 standard deviation below the mean for benefit finding.

Within individuals, increases in gastrointestinal symptoms predicted declines in PCS over time, whereas benefit finding showed no main effect. However, a significant interaction indicated that benefit finding moderated the impact of gastrointestinal symptoms on PCS. This interaction is portrayed in Figure 1. At high levels of benefit finding, the adverse impact of gastrointestinal symptoms on PCS was attenuated.

### 3.4. HLM Model for MCS

Table 2 (second column) presents a summary of the results of the analysis predicting MCS scores, controlling for age, gender, income, and cancer stage. Age was positively associated with MCS, with older individuals reporting higher MCS scores.

**Figure 2:**
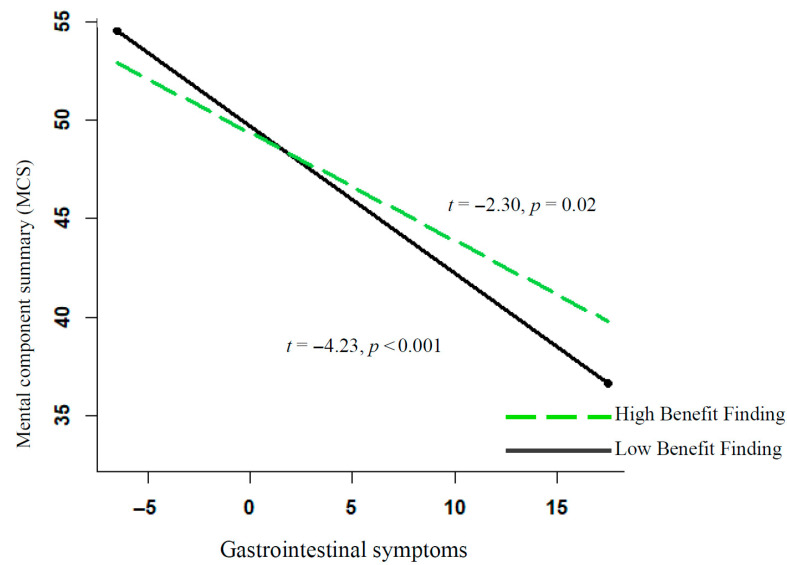
Moderation effect of benefit finding on gastrointestinal symptoms and MCS Score. Depicted are slopes for 1 standard deviation above and 1 standard deviation below the mean for benefit finding.

Within individuals, increases in gastrointestinal symptoms predicted declines in MCS over time, whereas benefit finding showed no main effect. However, a significant interaction indicated that benefit finding moderated the impact of gastrointestinal symptoms on MCS. This interaction is portrayed in Figure 2. At high levels of benefit finding, the adverse impact of gastrointestinal symptoms on MCS was attenuated.

## 4. Discussion

Using a two-year, multilevel framework, we demonstrated that within-person increases in gastrointestinal-symptom distress predicted contemporaneous declines in both physical and mental domains of HRQOL. While benefit finding showed no direct association with HRQOL, benefit finding moderated the symptom-HRQOL link, such that the deleterious impact of symptom distress was weakest among individuals reporting higher benefit finding. These results suggest that benefit finding functions as a context-dependent resource rather than a universally protective factor [43]. The moderation effects (effect sizes = 0.23) represent a small-to-moderate magnitude, indicating that, in clinical terms, patients who report greater benefit finding may be somewhat better equipped to maintain quality of life despite symptom burden.

To our knowledge, this is the first study that examined the relationship between gastrointestinal symptoms and HRQOL moderated by benefit finding and has used a longitudinal multiple-assessment design to investigate changes in gastrointestinal symptoms and benefit finding and the relationship over time with HRQOL. The study revealed substantial within-individual variability over time in gastrointestinal symptoms, benefit finding, and HRQOL—exceeding between-person differences—underscoring the importance of a multiple-assessment design and examining within-person change.

The strong, negative coupling between fluctuations in symptom distress and HRQOL replicates a well-established pattern in colorectal and other cancers: patients who experience heavier symptom loads report poorer HRQOL and well-being over time [5,10,44]. Our longitudinal design rules out several person-level confounds, reinforcing the interpretation that changes in somatic discomfort drive parallel changes in perceived quality of life.

Of greater interest was the interaction between gastrointestinal symptoms and benefit finding in predicting PCS and MCS. The interactions indicate that benefit finding moderated the impact of gastrointestinal symptoms on PCS and MCS. In individuals reporting higher benefit finding, the negative impact of gastrointestinal symptom distress on PCS and MCS is reduced. Consistent with the meta-analytic review, benefit finding showed no main-effect relationship with global HRQOL [12,26,45]. Null main effects are common; benefit finding tends to correlate more robustly with positive affect than with broad functional outcomes. The moderation we observed, however, aligns with a growing body of work suggesting that benefit finding is most consequential under conditions of elevated stress [18,43]. Our data extend these observations by showing a similar buffering pattern for gastrointestinal symptom distress at the intra-individual level.

Several non-mutually exclusive mechanisms may explain the buffering effect. Benefit finding reflects successful reappraisal of the cancer experience, which can dampen negative emotional reactions to aversive somatic cues and preserve engagement in valued activities [19]. Moreover, survivors who perceive benefits (e.g., closer relationships, greater appreciation of life) may mobilize social and psychological resources that offset the functional losses imposed by symptoms [29]. Positive cognitions can broaden attention and foster flexible coping, counteracting the narrowing effects of pain and fatigue [46]. Similarly, a recent study showed that positive meaning-making blunted the impact of multiple symptoms on HRQOL among CRC survivors [25]. Another study found that meaning-oriented cognitive processes can mitigate pain catastrophizing in individuals with gastrointestinal cancer [44]. Future studies incorporating ecological momentary assessment and psychophysiological markers could test these pathways directly.

### 4.1. Clinical Implications

From a therapeutic perspective, the evidence reported here suggests that benefit finding may have the potential to promote better adjustment to cancer. Interventions that facilitate benefit finding—such as benefit-finding writing, meaning-centered therapy, or brief cognitive-behavioral exercises—may be particularly useful for patients with high symptom burden [27,47]. Importantly, our findings caution against one-size-fits-all approaches: benefit-finding promotion may offer little added value for survivors whose symptom distress is low or well-controlled. Screening for both symptom severity and perceived benefits could help clinicians tailor supportive-care referrals.

### 4.2. Limitations

Focusing on CRC survivors, the study’s small, culturally homogeneous sample limits generalizability; replication in larger, more diverse cohorts and across other cancer types is warranted. It is important to note that the measure of physical symptoms in our study specifically focuses on gastrointestinal symptoms associated with CRC.

Although gastrointestinal symptom distress is common and may result from both disease and treatment [10,33], focusing solely on this domain neglects other common symptom dimensions, such as fatigue, sleep disturbance, and emotional distress [3,4,5]. Therefore, the scale may not fully capture the broader symptom burden experienced by CRC survivors. Chronic postsurgical pain is also common after CRC treatment and meaningfully degrades HRQOL, highlighting the need for proactive pain assessment and multidisciplinary management [48]. Anastomotic leakage can cause considerable distress, as it imposes substantial clinical and economic burdens and is associated with poorer HRQOL, particularly during the first postoperative year [49]. Future studies should include these symptoms to capture the full scope of survivorship burden.

We acknowledge that beyond symptom distress and psychosocial moderators, several clinical and surgical factors plausibly shape HRQOL trajectories after CRC surgery. In our study, age was positively correlated with MCS but negatively correlated with gastrointestinal symptoms. Early-onset CRC often presents with distinct mutational profiles and more advanced stages, which may confer different survivorship needs and recovery patterns relative to later-onset disease [50]. Postoperative venous thromboembolism (VTE) remains a salient complication; adherence to prophylaxis is associated with reduced VTE incidence after CRC surgery and may yield downstream benefits for functional recovery and quality of life [51]. In our study, no participants experienced VTE; therefore, this issue was not addressed further. Although this study did not assess surgical variables, such factors are important determinants of HRQOL, albeit not without controversy. Surgical technique and extent may influence recovery: robotic reduced-port approaches have not consistently demonstrated superior patient-reported outcomes compared with conventional laparoscopy (and may incur higher costs in some settings), whereas minimally invasive multivisceral resections can reduce short-term morbidity relative to open surgery in selected patients [52]. The presence and timing of stoma closure are also relevant, as delayed ileostomy reversal has been associated with lower HRQOL and increased psychological burden [53].

## 5. Conclusions

Despite these limitations, the present study extended the literature on the adaptive role of benefit finding. We examined several time points in the post-cancer trajectory and possible intra-individual variability that might influence the relationship of symptom distress, benefit finding, and HRQOL. ICCs showed that most variability in quality-of-life scores, gastrointestinal symptoms, and benefit finding arose from within-person fluctuations rather than between-person differences, reinforcing the need to examine temporal change and the contextual nature of these variables. Based on these accounts, our findings help to illustrate to whom benefit finding mitigates distress and to whom it fails to do so. Symptom management remains central to maintaining HRQOL, yet our results suggest that cultivating a sense of benefit from the cancer experience can soften—though not eliminate—the negative consequences of symptom flare-ups. Integrating benefit-finding strategies into supportive-care programs might therefore yield incremental gains, especially for survivors confronting persistent or worsening physical symptoms.

## Figures and Tables

**Table 1:** Means scores on all variables across time.

		T1	T2	T3
		**(*n* = 70)**	**(*n* = 59)**	**(*n* = 56)**
	**Scores Range**	***M* (*SD*)**	***M* (*SD*)**	***M* (*SD*)**
Gastrointestinal symptoms	0–24	6.24 (4.84)	6.19 (4.96)	5.59 (4.02)
Benefit Finding	6–30	19.14 (4.79)	19.61 (4.55)	19.77 (5.00)
Physical component summary (PCS) scores	0–100	50 (8.66)	50 (8.76)	50 (8.66)
Mental component summary (MCS) scores	0–100	50 (10.15)	50 (9.83)	50 (8.47)

Note: of the 73 participants enrolled at baseline, 56 remained at the final assessment, yielding an attrition rate of 23%. Attrition was primarily attributable to health-related difficulties, physical limitations, treatment transfer, and loss to follow-up.

**Table 2:** Results of the hierarchical linear modeling predicting PCS and MCS.

	Physical Component Summary (PCS)				Mental Component Summary (MCS)			
Predictors	*b*	*SE*	*t*	Effect Size *r*	*b*	*SE*	*t*	Effect Size *r*
Within-person effect								
Gastrointestinal symptoms	−0.70	0.13	−5.26 ***	0.52	−0.55	0.13	−4.18 ***	0.44
Benefit finding	−0.07	0.12	−0.58	0.07	−0.07	0.12	−0.54	0.06
Symptoms * benefit finding	0.04	0.02	1.98 *	0.23	0.04	0.02	2.00 *	0.23
Time	−0.05	0.58	0.93	0.11	−0.03	0.53	0.95	0.11
Between-person effect								
Age	0.04	0.07	0.59	0.07	0.23	0.09	2.70 **	0.30
Gender	−1.06	1.47	−0.72	0.08	0.81	1.83	0.44	0.05
Income	2.04	0.72	2.83 **	0.31	0.86	0.90	0.95	0.11
Stage	0.34	0.49	−0.72	0.08	0.13	0.61	0.21	0.02
Constant	50.60	1.30	38.78 ***		49.37	1.55	31.76 ***	

Note: *N* = 73; *n* = 219 across three time points.* *p* < 0.05, ** *p* < 0.01, *** *p* < 0.001.

## Data Availability

The raw data supporting the conclusions of this article will be made available by the authors on request.
